# Comparison of Two Dietary Supplements for Treatment of Uric Acid Renal Lithiasis: Citrate vs. Citrate + Theobromine

**DOI:** 10.3390/nu12072012

**Published:** 2020-07-07

**Authors:** Yumaira Hernandez, Antonia Costa-Bauza, Paula Calvó, Joan Benejam, Pilar Sanchis, Felix Grases

**Affiliations:** 1 Urology Service of Manacor Hospital, 07500 Balearic Islands, Spain; hernandezyumaira@gmail.com (Y.H.); jmbenejam@hmanacor.org (J.B.); 2 Laboratory of Renal Lithiasis Research, University Institute of Health Sciences Research (IUNICS-IdISBa), University of Balearic Islands, 07122 Palma de Mallorca, Spain; paula.calvo@uib.es (P.C.); pilar.sanchis@uib.es (P.S.); fgrases@uib.es (F.G.)

**Keywords:** uric acid, urolithiasis, theobromine, dietary treatment

## Abstract

Background. Uric acid (UA) renal lithiasis has a high rate of recurrence and a prevalence ranging from 10% and 15%, depending on the population. The most important etiological factor is persistence of urinary pH below 5.5 and one of the most common treatments is alkalization with citrate. Recent studies demonstrated that theobromine, which is abundant in chocolate and cocoa, is a potent inhibitor of UA crystallization. Aim. The aim was to compare the efficacy of citrate versus citrate + theobromine as treatment for UA lithiasis. Methods. This randomized cross-over trial investigated the efficacy of two treatments in 47 patients with UA renal lithiasis. Urine volume, pH, UA excretion, theobromine excretion, and risk of UA crystallization (RUAC) at baseline and at the end of each intervention period were measured. Results. Each treatment significantly reduced the risk of UA crystallization compared to basal values. The RUAC after citrate + theobromine was lower than the RUAC after citrate, although this difference was not statistically significant. Conclusion. The combined consumption of citrate and theobromine may be a promising strategy for the prevention of UA kidney stones.

## 1. Introduction

Uric acid (UA) renal lithiasis has a high rate of recurrence [1,2], an incidence that increases with age [3,4], and an overall prevalence of 10% and 15% depending on the population [5,6]. The major etiological risk factor is persistence of a urinary pH below 5.5 [7,8,9,10]. Alkalizing treatment with dietary citrate is currently one of the most common treatments for UA renal lithiasis [11,12,13].

We recently demonstrated that dietary theobromine is a potent inhibitor of UA crystallization in humans [13,14]. Theobromine is abundant in chocolate and cocoa [15]. Chemically, it is known as 3, 7-dimethylxanthine and, along with caffeine and theophylline, is in the xanthine family of alkaloids. However, it has weaker effects on the central nervous system than caffeine [16]. Theobromine, due to its unique structural and chemical characteristics, has a potent inhibitory effect on the crystallization of UA [13] and this effect is clinically significant when its urinary concentration is 15 mg/L or higher. It is thus a novel inhibitor of UA crystallization with potential clinical applications. About 20% of orally consumed theobromine is excreted in the urine [17,18]. Individuals who do not consume theobromine (or chocolate) have low, though detectable, urinary levels of the compound, since it also comes from the metabolism of caffeine [19]. Considering that dark chocolate contains 1% to 4% theobromine, the recommended amount would correspond to a daily intake of approximately 10 g of dark chocolate. This amount is obviously acceptable for most individuals, except for those with diabetes, obesity, or metabolic syndrome. Unfortunately, individuals with precisely these conditions are more prone to UA stones. Chocolate consumption is also inadvisable for individuals with oxalocalcic lithiasis because chocolate is very rich in oxalate. For this reason, consumption of cocoa extract rather than chocolate may be more widely acceptable.

This paper presents a comparative study of the treatment of UA renal lithiasis with citrate versus citrate + theobromine.

## 2. Materials and Methods

### 2.1. Participants

This double-blind, randomized, cross-over study recruited 54 volunteer patients at the Urology Service of the Manacor Hospital (Balearic Islands, Spain) who had previous UA renal lithiasis or calcium oxalate monohydrate/UA renal lithiasis.

Patients were excluded if they were older than 75 years; had a serum creatinine level above 0.113 mmol/L; had hyperuricemia and/or were taking a diuretic, allopurinol, febuxostat, or other hyperuricemia medication; had an allergy to chocolate or theobromine; were pregnant or lactating; or had an intestinal obstruction. The randomized controlled trials’ registration number is NCT03483532.

### 2.2. Treatments

**Figure 1:**
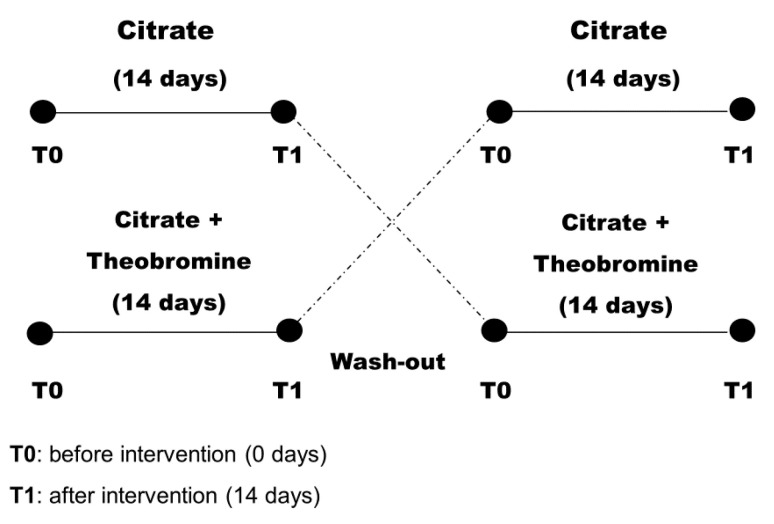
Design of the randomized crossover study of patients (*n* = 47).

All subjects received oral and written information about their treatments. Subjects were randomly assigned to citrate group or a citrate + theobromine group for 14 days. Then, after a 7-day washout period, all subjects were switched to the alternate treatment for an additional 14 days (Figure 1). Patients were asked not to change their use of medications during the study period.

All patients received two tablets, one at breakfast and the other at dinner. The citrate tablet contained 0.653 mmol K-Cit + 0.933 mmol Mg-Cit. The citrate + theobromine tablet contained 0.653 mmol K-Cit + 0.933 mmol Mg-Cit + 0.333 mmol theobromine extract. Tablets were supplied by Devicare S.L. (Spain). After randomization, there was no blinding of the researchers administering the interventions, but there was blinding of the study participants and the researchers assessing outcomes.

Each patient was monitored weekly to ensure the treatment was followed. The dietician also checked for compliance (consumption of at least 80% of pills) at every visit by counting the remaining pills. The basal characteristics of all patients were recorded (Table 1).

### 2.3. Outcome Parameters

Clinical histories were extracted from the electronic medical records. Samples of 24 h urine were collected at baseline to verify satisfaction of the admission criteria and 2 h urine collected after overnight fasting at baseline and after each stage of treatment. Treatment efficacy was evaluated by a test specially designed to measure the ability of urine to inhibit the crystallization of UA (a risk of UA crystallization (RUAC) test) [14] and urinary theobromine was measured using HPLC (High Performance Liquid Chromatography), as previously described [19].

Urine volume, pH, creatinine, UA, and RUAC test results were determined for each sample. Urinary pH was measured using a Crison pH-meter GLP22, creatinine with the kinetic Jaffe method, and UA was measured using the uricase method.

**Figure 2:**
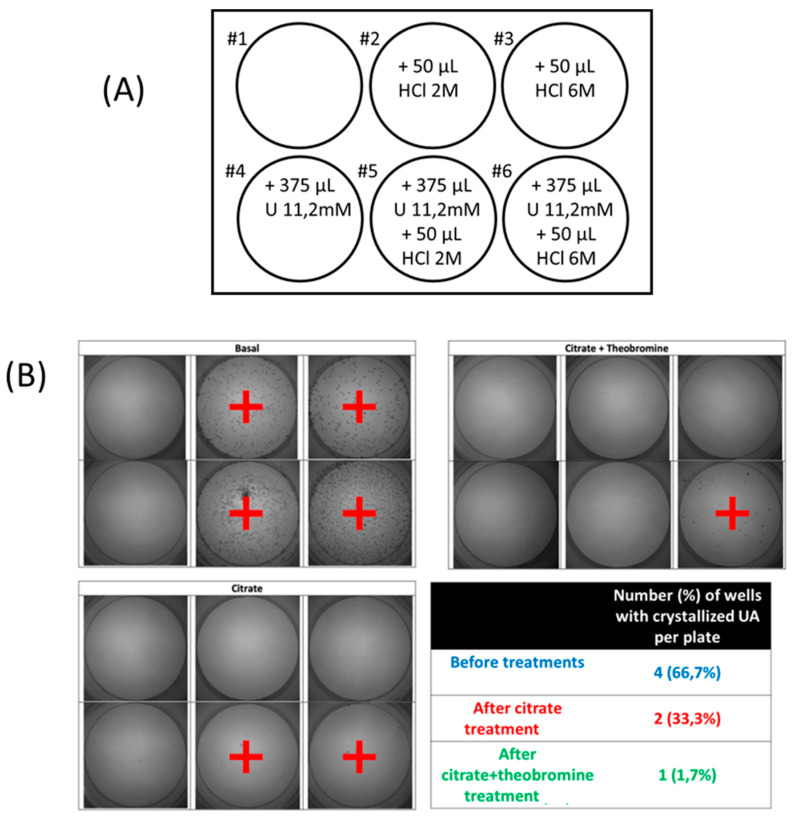
(**A**) Protocol board diagram to establish the risk of crystallization or uric acid (RUAC). Six wells of a 12-well plate were used for each sample of urine, with each well containing 5 mL of urine and different amounts of HCL and urate. Well #1 had urine alone, well #2 had urine and 50 µL of 2 M HCl (0.1 mmoL H^+^), well #3 had urine and 50 µL of 6 M HCl (0.3 mmol H^+^), well #4 had urine and 4.2 µmol urate (375 µL of 11.2 mM urate solution), well #5 had urine, 0.1 mmoL H^+^ and 4.2 µmol urate, and well #6 had urine, 50 mL 0.3 mmol H^+^ and 4.2 µmol urate. (**B**). Images of RUAC test in which wells with uric acid crystals have been marked with +, and results of one representative individual using RUAC protocol before and after each treatment.

The RUAC test was carried out in polystyrene non-treated well plates (Corning, NY, USA). First, 5 mL of each urine sample was added to each of the 6 wells. The first well had no additions. Hydrochloric acid and/or UA were added to the other wells to promote the crystallization of UA (Figure 2A). The 6 wells were kept at room temperature for 24 h, after which the urine was removed. The result was determined by the number of wells in which there were UA crystals, and ranged from 0 (crystals in no wells) to 6 (crystals in all wells) (Figure 2B). For data analysis, a “low RUAC” score was considered to be 4 or less and a “high RUAC” score was considered to be 5 or 6.

Type 2 diabetes was diagnosed if subjects presented with fasting serum glucose of at least 7.0 mmol/L and/or glycated hemoglobin of at least 6.5% [20]. Blood pressure was measured 3 times consecutively after a 5 min rest while the subject was sitting quietly. The average of the second and third measurements was recorded. Patients using anti-hypertensive drugs and those with systolic blood pressure of 140 mmHg or more and/or diastolic blood pressure of 90 mmHg or more were categorized as having hypertension [21]. Dyslipidemia was defined as the presence of one of the following: LDL cholesterol level of 100 mg/dL or more, HDL cholesterol level below 40 mg/dL (men) or 50 mg/dL (women), triglycerides of 150 mg/dL or more, or use of a lipid-lowering drug [22].

### 2.4. Statistical Analysis

Data are presented as means ± standard deviations or numbers (percentages). Intra-group differences before (T0) vs. after (T1) the intervention were evaluated using a paired-samples *t*-test or Wilcoxon’s signed-rank paired test for continuous parameters and McNemar test for categorical parameters. Inter-group comparisons after the intervention were assessed using analysis of covariance with adjustment for changes in continuous parameters according to baseline values. A two-tailed *p*-value less than 0.05 was considered statistically significant. Statistical analyses were performed using SPSS version 23.0 (SPSS Inc., Chicago, IL, USA).

### 2.5. Ethical Considerations

The study design was approved by the Research Committee of Hospital Manacor and Research Ethics Committee of Balearic Islands (CEI-IB) (IB3414). All patients provided written informed consent before participation.

## 3. Results

Forty-seven patients (5 females and 42 males) completed both treatments in this cross-over clinical study (Table 1). The mean age was 60.4 ± 10.1 years and the mean body mass index (BMI) was 30.2 ± 4.7. Ten patients (21.3%) had diabetes, 22 (46.8%) had hypertension, 16 (34.0%) had dyslipidemia, and 22 patients (46.8%) were obese (BMI > 30).

**Figure 3:**
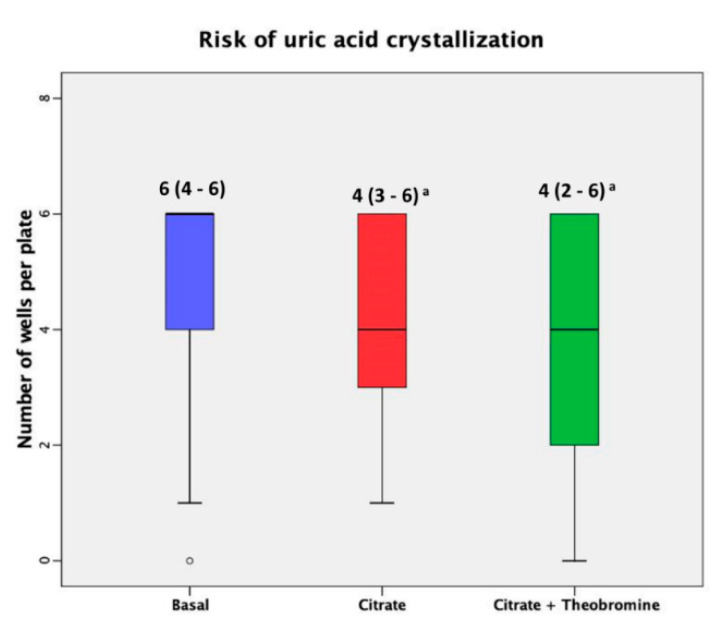
Risk of crystallization of uric acid at baseline and after both treatments. Median (interquartile ranges) of the number of crystallized wells before and after both dietary interventions. The dark lines in the boxes are the medians. The bottom and the top of the box indicate the 25th and the 75th percentiles, respectively. These values are indicated on each box. The T-bars that extend from the boxes are the inner fences. The point is an outlier. (a) *p* < 0.001 vs. basal value.

Table 2 shows the urinary parameters at baseline and after each treatment and Figure 3 shows the results of the RUAC test before and after each treatment. These results indicate that citrate treatment and citrate + theobromine treatment led to significantly lower RUAC scores than at baseline (*p* < 0.01 and *p* < 0.001, respectively).

**Figure 4:**
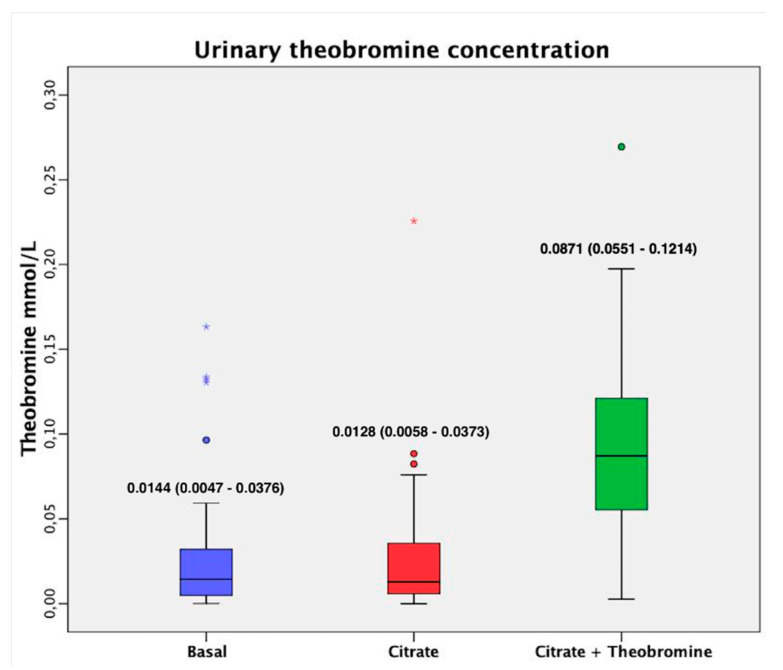
Urinary theobromine concentration before and after both treatments. Values are expressed as median (interquartile range). The dark lines in the boxes are the medians. The bottom and the top of the box indicate the 25th and the 75th percentiles, respectively. The T-bars that extend from the boxes are the inner fences. These values are indicated on each box. The points are outliers and the asterisks are extreme outliers. (1) *p* < 0.001 vs. basal value; (2) *p* < 0.001 vs. after citrate treatment.

As expected, the urine of patients who received citrate + theobromine had a significantly higher concentration of theobromine than patients at baseline and patients who received citrate alone (Figure 4, *p* < 0.001). No patients had to discontinue participation in the study due to adverse events.

## 4. Discussion

Considering the solubility of UA in aqueous media, urine with a normal UA content only leads to UA lithiasis when the pH is below 5.5 [23]. Thus, citrate is the therapy of choice for these patients due to its alkalizing effects on urine. However, a problem with citrate therapy is that high doses can increase the urinary pH above 6.2. This can induce the formation of insoluble calcium phosphate salts, such as hydroxyapatite or brushite, which can give rise to mixed stones and the generation of nephrocalcinosis. When hyperuricosuria is present, deposits of sodium and/or potassium urate can also develop. In fact, previous studies reported mixed stones of UA and hydroxyapatite [24]. Obviously, if the citrate treatment does not sufficiently increase the urinary pH, then development of UA stones may still occur. For this reason, the combination of a urinary basifying agent, such as citrate, with an inhibitor of UA crystallization, such as theobromine, may be an effective treatment. Such a treatment would provide sufficient alkalization with lower doses of citrate, thus preventing excessive basification and avoiding UA crystal formation.

Our results showed that treatment with citrate or citrate + theobromine significantly reduced the risk of UA lithiasis relative to baseline (Figure 3). Although the efficacy of citrate + theobromine treatment was better, this did not reach statistical significance, possibly because the citrate dose alone was very effective in the present case and there was no excessive increase in pH probably because of the low dose that was administered. Using a low dose of citrate, in addition to preventing the crystallization of insoluble calcium phosphates, leads to fewer undesirable side effects, such as gastrointestinal discomfort, and also reduces the supply of potassium, which may be relevant in patients with kidney failure or heart disease. We also found that the urine of patients treated with citrate + theobromine had a significantly higher concentration of theobromine than at baseline and after treatment with citrate alone (p < 0.001); the levels at baseline and after treatment with citrate alone were not significantly different. Thus, the theobromine urinary concentrations observed are in the low limit of efficacy (0.083 mmol/L) [13]. Obviously, one might expect a greater effect using higher doses.

The exclusive use of theobromine could be considered for the treatment of patients with UA lithiasis. However, it is interesting to take into account that the combined use of citrate and theobromine has a synergistic effect, because small increases in urinary pH greatly enhance the effects of theobromine as an inhibitor of UA crystallization [13] and reduces UA supersaturation.

This is the first study of patients with UA lithiasis to evaluate the effects of a combination of citrate and theobromine. A limitation is that we only examined a small number of patients from a single center. A study with a larger and more diverse sample is necessary for confirmation.

## 5. Conclusions

The combined use of citrate and theobromine may be an effective strategy to prevent the formation of UA kidney stones. The inhibitory effect of theobromine on UA crystallization allows use of lower doses of citrate and thereby prevents the generation of high urinary pH that could induce the formation of insoluble calcium phosphates. The combined treatment also provides protection against UA crystallization if there is an excessive drop in urinary pH.

## Figures and Tables

**Table 1:** Baseline characteristics of patients (*n* = 47). Each value is given as mean ± SD or number (percentage).

Baseline Characteristics of Patients (*n* = 47)		
Gender (male)	42	(89.4%)
Age (years)	60.4	±10.1
BMI (kg/m^2^)	30.2	±4.7
Systolic blood pressure (mmHg)	143	±18
Diastolic blood pressure (mmHg)	87	±10
Type of calculi		
UA	34	(72.3%)
UA/COM	12	(25.5%)
UA/AU	1	(2.1%)
Type 2 diabetes mellitus	10	(21.3%)
Hypertension	22	(46.8%)
Dyslipidemia	16	(34.0%)
BMI categories		
25–30 kg/m^2^	18	(38.3%)
30–35 kg/m^2^	14	(29.8%)
>35 kg/m^2^	7	(14.9%)

UA: uric acid; COM: calcium oxalate monohydrate; AU: ammonium urate.

**Table 2:** Values in parameters for 2 h urine before and after the intervention (14 days). Values are given as mean ± SD. Intra-group analysis (before vs. treatment) used a paired-sample Wilcoxon signed-rank test or paired-samples *t*-test to determine the significance of differences. Inter-group analysis (citrate vs. citrate + theobromine) used analysis of covariances and comparison between groups after adjusting for baseline levels to determine the significance of differences.

Parameter	Baseline	After Citrate Treatment		After Citrate + Theobromine		InterG *p*-Value
	Mean ± SD	Mean ± SD	IntraG *p*-Value	Mean ± SD	IntraG *p*-Value	
Volume (L)	0.078 ± 0.023	0.083 ± 0.044	0.674	0.08 ± 0.03	0.767	0.311
pH	5.45 ± 0.47	5.45 ± 0.44	0.879	5.49 ± 0.46	0.620	0.672
Creatinine (mmol/L)	9.8 ± 3.3	9.5 ± 3.9	0.388	9.9 ± 3.6	0.532	0.514
Uric acid (mmol/L)	2.8 ± 1.1	2.7 ± 1.3	0.536	2.9 ± 1.4	0.400	0.460
Theobromine (mmol/L)	0.029 ± 0.039	0.036 ± 0.070	0.604	0.102 ± 0.086	<0.001	<0.001

The percentage of patients with low RUAC scores (≤ 4) and high RUAC scores (> 4) before and after each treatment is lower for patients with high RUAC scores after citrate (44.7%) and after citrate + theobromine (38.3%) than at baseline (63.8%). Comparison of the two treatments indicates that citrate + theobromine leads to a lower median RUAC score (Figure 3, *p* = 0.194) and a lower percentage of patients with high RUAC scores (*p* = 0.676), although these differences are not significant.
